# Scalable time-constrained planning of multi-robot systems

**DOI:** 10.1007/s10514-020-09937-6

**Published:** 2020-07-31

**Authors:** Alexandros Nikou, Shahab Heshmati-alamdari, Dimos V. Dimarogonas

**Affiliations:** Division of Decision and Control, School of Electrical Engineering and Computer Science, KTH Royal Institute of Technology, 100 44 Stockholm, Sweden

**Keywords:** Multi-robot systems, Cooperative control, Decentralized control, Abstractions, Metric interval temporal logic (MITL), Nonlinear model predictive control (NMPC), Robust control

## Abstract

This paper presents a scalable procedure for time-constrained planning of a class of uncertain nonlinear multi-robot systems. In particular, we consider *N* robotic agents operating in a workspace which contains regions of interest (RoI), in which atomic propositions for each robot are assigned. The main goal is to design decentralized and robust control laws so that each robot meets an individual high-level specification given as a metric interval temporal logic (MITL), while using only local information based on a limited sensing radius. Furthermore, the robots need to fulfill certain desired transient constraints such as collision avoidance between them. The controllers, which guarantee the transition between regions, consist of two terms: a nominal control input, which is computed online and is the solution of a decentralized finite-horizon optimal control problem (DFHOCP); and an additive state feedback law which is computed offline and guarantees that the real trajectories of the system will belong to a hyper-tube centered along the nominal trajectory. The controllers serve as actions for the individual weighted transition system (WTS) of each robot, and the time duration required for the transition between regions is modeled by a weight. The DFHOCP is solved at every sampling time by each robot and then necessary information is exchanged between neighboring robots. The proposed approach is scalable since it does not require a product computation among the WTS of the robots. The proposed framework is experimentally tested and the results show that the proposed framework is promising for solving real-life robotic as well as industrial applications.

## Introduction

Over the last few years, the field of control of multi-robot systems under high-level specifications has been gaining significant attention (Wongpiromsarn et al. 2009; Nikou 2019; Kantaros and Zavlanos 2016; Hasanbeig et al. 2019; Pant et al. 2018, 2019; Raman et al. 2014). Applications arise in the fields of autonomous driving, industrial work by autonomously operating robot systems, indoor transportation in warehouses etc (Ulusoy et al. July 2013; Xu et al. 2019). The qualitative specification language that has primarily been used to express the high-level tasks is linear temporal logic (LTL) (see, e.g., Fainekos et al. February 2009; Fang et al. 2018). In practical applications, however, the desired tasks need to be accomplished within certain quantitative time bounds by the robots.

A suitable temporal logic for dealing with tasks that are required to be completed within certain time bounds is the metric interval temporal logic (MITL). MITL has been originally proposed in Alur and Dill (1994) and has been used in control synthesis frameworks in Nikou et al. (2016). Given a robot dynamics and an MITL formula, the control design procedure is the following: first, the robot dynamics are abstracted into a weighted transition system (WTS), in which the time duration for navigating between states is modeled by a weight in the WTS (abstraction); second, an offline product between the WTS and an automaton that accepts the runs that satisfy the given formula is computed; and third, once an accepting run in the product is found, it maps into a sequence of feedback control laws of the robot dynamics.

Controller synthesis for multi-robot systems under MITL specifications has been investigated in Nikou et al. (2017, (2018, (2017), Karaman and Frazzoli (2008). In our previous works (Nikou et al. 2017, 2018, 2017), the under consideration dynamics were first order and we considered actuation over each state of each agent. Additionally, a global product WTS of the individual WTSs of each robot was computed. Furthermore, no transient constraints between the agents are taken into consideration. In particular, the work (Nikou et al. 2017) can only handle multi-agent timed constrained planning in $\mathbb {R}^2$ dimension, which is usually not the case in real-life robotic applications. Authors in Karaman and Frazzoli (2008) have addressed the vehicle routing problem (VHP), which is modeled as an optimization problem, that aims at finding the optimal set of routes for a fleet of vehicles to traverse, in order to deliver the load to a given set of customers. However, the dynamics of the agents were not taken into consideration. Moreover, issues such as control input saturation and robustness against disturbances were not considered. In the same context, none of the aforementioned works deal with real-time experimental validation of the corresponding proposed frameworks.

**Fig. 1:**
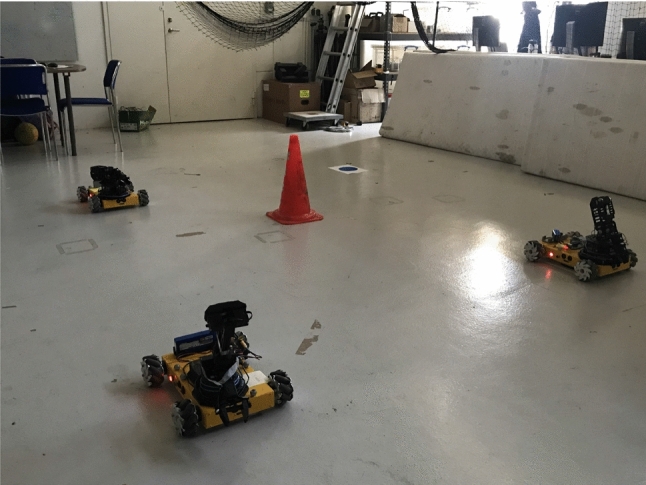
The experimental setup demonstrating the proposed framework. Three Nexus 10011 mobile robots, in the workspace of Smart Mobility Lab (SML) (n.d.) that contains 5 RoI

Motivated by the aforementioned, in this work, we aim to address the latter issues. In particular, we deal with nonlinear dynamics in $\mathbb {R}^n$ with input constraints and external uncertainties/disturbances. Then, by assigning an MITL formula to each agent, we provide decentralized feedback control laws that guarantee robust transitions between neighboring agents under transient constraints. The controllers consist of two terms: a nominal control input, which is computed online and is the solution of a decentralized finite-horizon optimal control problem (DFHOCP); and an additive state feedback law which is computed offline and guarantees that the real trajectories of the system will belong to a hyper-tube centered along the nominal trajectory. More specifically, the online part is responsible for minimizing the error and the control input effort in order for the robot to be navigated between RoI, while transient constraints and control input saturation are satisfied. The second part is introduced in order to guarantee that while the DFHOCP is solved for the nominal dynamics, the controller compensates for the uncertain part due to external disturbances and keeps the trajectory of the robot bounded inside a tube. Furthermore, an algorithm that computes the runs of each agent that in turn map into continuous control laws and provably satisfy the MITL formulas is provided. These control laws correspond to the transitions indicated above. The proposed scheme is experimentally validated in our lab facilities with a group of Nexus robots (see Fig. 1). Moreover, the proposed approach is scalable since does not require a product computation among the WTS of the agents.

The idea of avoiding the global product between the agents lies in the fact that we address the multi-agent coupling with the low-level continuous time control design. More specifically, we exploit the inherent advantage of NMPC with reference to other control techniques: we capture the coupling constraints through the hard constraints of each agent by assuming communication capabilities between the agents. In the same context, all the algorithmic computations are performed offline and the robots are executing online only a sequence of a control actions that are the outcome of the planner. Thus, the latter leads to a framework that is scalable with the number of agents. Our contribution is thus a fully automated framework for a general class of uncertain multi-robot systems consisting of both constructing purely decentralized abstractions and conducting timed temporal logic planning with transient constraints in a scalable way.

This paper is structured as follows. In Sect. 2 a description of the necessary mathematical tools, the notations and the definitions are given. Section 3 provides the modeling of the proposed framework along with the formal problem statement. Section 4 discusses the technical details of the solution, while Sect. 5 is devoted to a real-time experiment demonstrating the proposed approach. Finally, conclusions and future research directions are discussed in Sect. 6.

## Notation and preliminaries

In this section, the notation that will be used hereafter as well as the necessary mathematical background for nonlinear control systems and formal verification are provided.

### Notation

The set of positive integers, positive rational numbers and real numbers are denoted by $\mathbb {N}$, $\mathbb {Q}_{+}$, and $\mathbb {R}$, respectively. Given a set *S*, we denote by $|S|$ its cardinality, by $S^n = S \times \dots \times S$ its *n*-fold Cartesian product, and by $2^S$ the set of all its subsets; $\Vert y\Vert _2 {:}{=}\sqrt{y^\top y}$ and $\Vert y\Vert _{M} {:}{=}\sqrt{y^\top M y}$, $M \ge 0$ stand for the Euclidean and the weighted norm of a vector $y \in \mathbb {R}^n$, respectively; $\lambda _{\text {min}}(M)$ stands for the minimum absolute value of the real part of the eigenvalues of $M \in \mathbb {R}^{n \times n}$; $I_n \in \mathbb {R}^{n \times n}$ and $0_{m \times n} \in \mathbb {R}^{m \times n}$ are the identity matrix and the $m \times n$ matrix with all entries zeros, respectively. A $n \times n$ symmetric real matrix *M* is said to be positive semidefinite ($M \ge 0$) if $x^\top M x \ge 0$ for every $x \in \mathbb {R}^n$. The notation $\mathrm{diag}\{M_1, \dots , M_n\}$ stands for the block diagonal matrix with the matrices $M_1$, $\dots$, $M_n$ in the main diagonal. The set $\mathcal {M}(\chi , \rho ) = \{y \in \mathbb {R}^n: \Vert y-\chi \Vert _2 \le \rho \},$ represents the *n*-dimensional ball with center $\chi \in \mathbb {R}^{n}$ and radius $\rho \in \mathbb {R}_{> 0}$. It should be noticed that, throughout this manuscript, the nominal signals and the magnitude of upper bounds of signals are denoted with $\overline{\cdot }$, $\widetilde{\cdot }$, respectively. Tables 1 and 2 show a list of symbols and a list of acronyms, respectively, that are used in this manuscript.

**Table 1:** List of symbols

$\mathbb {N}, \mathbb {Q}, \mathbb {R}$	Natural, rational & real numbers
$[N] {:}{=}\{1, \dots , N\}$	Labeling set of robots
$[Z] {:}{=}\{1, \dots , Z\}$	Labeling set of RoI
$\mathcal {R} = \bigcup _{z \in [Z]} R_z$	Union of RoI
$\mathcal {W}$	The workspace of the robots
*L*	Lipschitz constant
$\mathcal {T}_i$, $\mathcal {A}_i$, $\widetilde{\mathcal {T}}_i$	WTS, TBA, product WTS of robot *i*
$(S, S^{\text {init}}, \text {Act}, \longrightarrow , \mathfrak {t}, \varGamma , \mathcal {L})$	(states, init. states, actions, transition relation, weight, atomic propos., labeling function)
$(Q, Q^{\text {init}}, \text {CL}, \mathrm{Inv}, E, \text {FS})$	(states, init. states, clocks, invariance, accepted states)
$x_i, v_i, u_i$	position/orientation, velocity and control input of robot
$e_i = x_i - x_{i, d}$	Error signal to be minimized through DFHOCP
$\xi _i = [e_i, v_i]^\top$	stack vector
$f_i, G_i, \delta _i$	Dynamic model of robot *i* and disturbance $\delta _i$
$\mathfrak {e}_i = e_i - \overline{e}_i, \mathfrak {v}_i = v_i - \overline{v}_i$	Errors between real and nominal signals (state and velocity respectively)
$\varOmega _{i,1}, \varOmega _{i,2}$	Invariant tubes for the errors $\mathfrak {e}_i$, $\mathfrak {v}_i$
$P_i, Q_i, R_i$	Positive definite weight matrices used in DFHOCP
$\mathcal {E}_i, \mathcal {V}_i, \mathcal {U}_i, \mathcal {F}_i$	State constraints, velocity constraints, control input constraints, terminal set of DFHOCP
*T*, *h*	Prediction horizon and constant sampling time period
$k_i, \rho _i$	Tube gains
$r^t, w^t$	Timed run & timed word
$\tau (l), l \ge 0$	Time stamp at position $l \ge 0$
$\mathcal {M}(x_i, \mathfrak {r}_i)$	A ball centered at $x_i$, radius $\mathfrak {r}_i$ covering robot *i*
$d_i, \mathcal {G}_i(t)$	Sensing radius & set of neighbors of robot *i* at time *t*
$\Diamond _I, \square _I, \mathcal {U}_I$	Eventually, always and until MITL operators

**Table 2:** List of acronyms

NMPC	Nonlinear model predictive control
RoI	Regions of interest
DFHOCP	Decentralized finite horizon
	Optimal control problem
TBA	Timed Büchi automaton
WTS	Weighted transition system
MITL	Metric interval temporal logic
ISS	Input to state stability
RCI set	Robust control invariant set

#### Definition 1

Given the sets $\mathcal {S}_1$, $\mathcal {S}_2 \subseteq \mathbb {R}^n$, $\mathcal {S} \subseteq \mathbb {R}^m$ and the matrix $M \in \mathbb {R}^{n \times m}$, the *Minkowski addition*, the *Pontryagin difference* as well as the *matrix-set multiplication* are respectively defined by:

$$\begin{aligned} \mathcal {S}_1 \oplus \mathcal {S}_2&{:}{=}\{s_1 + s_2 : s_1 \in \mathcal {S}_1, s_2 \in \mathcal {S}_2\}, \\ \mathcal {S}_1 \ominus \mathcal {S}_2&{:}{=}\{s_1 : s_1+s_2 \in \mathcal {S}_1, \forall \ s_2 \in \mathcal {S}_2\}, \\ M \circ \mathcal {S}&{:}{=}\{m = Ms, \ s \in \mathcal {S}\}. \end{aligned}$$

#### Lemma 1

(Nikou and Dimarogonas 2019) For any constant $\rho > 0$, vectors $z_1$, $z_2 \in \mathbb {R}^n$ and matrix $P \in \mathbb {R}^{n \times n}$, $P > 0$ it holds that:

$$\begin{aligned} z_1 P z_2 \le \frac{1}{4 \rho } z_1^\top P z_1 + \rho z_2^\top P z_2. \end{aligned}$$

### Nonlinear control

#### Definition 2

Khalil (1996) A continuous function $\varvec{\alpha } : [0, a) \rightarrow \mathbb {R}_{\ge 0}$ belongs to *class* $\mathcal {K}$ if it is strictly increasing and $\varvec{\alpha } (0) = 0$. A continuous function $\varvec{\beta } : [0, a) \times \mathbb {R}_{\ge 0} \rightarrow \mathbb {R}_{\ge 0}$ belongs to *class* $\mathcal {KL}$ if: 1) for a fixed *s*, the mapping $\varvec{\beta }(r,s)$ belongs to class $\mathcal {K}$ with respect to *r*; 2) for a fixed *r*, the mapping $\varvec{\beta }(r,s)$ is strictly decreasing with respect to *s*; and it holds that: $\lim \nolimits _{s \rightarrow \infty } \varvec{\beta }(r,s) = 0$.

#### Definition 3

Yu et al. (2013) Consider a dynamical system:

$$\begin{aligned} \dot{x} = f(x,u,\delta ), \ x \in \mathcal {X}, \ u \in \mathcal {U}, \ \delta \in \mathcal {D}, \end{aligned}$$

with initial condition $x(0) \in \mathcal {X}$. A set $\mathcal {X}' \subseteq \mathcal {X}$ is a *Robust Control Invariant (RCI) set* for the system, if there exists a feedback control law $u {:}{=}\kappa (x) \in \mathcal {U}$, such that for all $x(0) \in \mathcal {X}'$ and for all disturbances $\delta \in \mathcal {D}$ it holds that $x(t) \in \mathcal {X}'$ for all $t \ge 0$, along every solution *x*(*t*).

#### Definition 4

(Khalil 1996, Def. 4.7, p. 175) A nonlinear system $\dot{x} = f(x, u, \delta )$, $x \in \mathcal {X}$, $u \in \mathcal {U}$, $\delta \in \mathcal {D}$ with initial condition $x(0) \in \mathcal {X}$ is said to be *Input-to-State Stable (ISS)* with respect to $\delta \in \mathcal {D}$, if there exist functions $\varvec{\beta } \in \mathcal {KL}$, $\varvec{\gamma } \in \mathcal {K}$ such that for any initial condition $x(0) \in \mathcal {X}$ and for any input $u(t) \in \mathcal {U}$, the solution *x*(*t*) exists for all $t \in \mathbb {R}_{\ge 0}$ and satisfies:

$$\begin{aligned} \Vert x(t)\Vert _2 \le \varvec{\beta }\big (\Vert x(0)\Vert _2,t\big ) + \varvec{\gamma } \left( \displaystyle \sup _{0 \le \mathfrak {s} \le t} \Vert \delta (\mathfrak {s})\Vert _2\right) . \end{aligned}$$

### Nonlinear model predictive control

NMPC Chen and Allgöwer (1998) is formulated as solving at each sampling time step an online finite horizon optimal control problem (FHOCP) subject to nonlinear system dynamics and constraints involving states and controls. Based on measurements obtained at each sampling time step, the controller predicts the dynamic behavior of the system over a predictive horizon in the future and determines the input such that a predetermined open-loop performance objective is minimized. In order to incorporate feedback, the optimal open-loop input is implemented only until the next sampling time step. Using the new system state at the next sampling time step, the whole procedure—prediction and optimization—is repeated.

### Formal verification

#### Definition 5

(Alur and Dill 1994) A *time sequence* $\tau = \tau (0) \tau (1) \ldots$ is an infinite sequence of time values $\tau (l) \in \mathbb {Q}_{+}$, satisfying the following properties: 1) Monotonicity: $\tau (l) < \tau (l+1)$ for all $l \in \mathbb {N}$; 2) Progress: For every $t \in \mathbb {Q}_{+}$, there exists $l \ge 1$, such that $\tau (l) > t$.

An *atomic proposition* *p* is a statement that is either True $(\top )$ or False $(\bot )$.

#### Definition 6

(Alur and Dill 1994) Let $\varGamma$ be a finite set of atomic propositions. A *timed word* *w* over the set $\varGamma$ is an infinite sequence $w^t = (w(0), \tau (0)) (w(1), \tau (1)) \ldots$ where *w*(0) *w*(1) $\ldots$ is an infinite word over the set $2^{\varGamma }$ and $\tau (0)$ $\tau (1)$ $\ldots$ is a time sequence with $\tau (l) \in \mathbb {Q}_{+}$, $l \in \mathbb {N}$.

#### Definition 7

A weighted transition system (*WTS*) is a tuple $(S, S^\text {init}, Act, \longrightarrow , \mathfrak {t}, \varGamma , \mathcal {L})$ where *S* is a finite set of states; $S^\text {init} \subseteq S$ is a set of initial states; *Act* is a set of actions; $\longrightarrow \subseteq S \times Act \times S$ is a transition relation; $\mathfrak {t}: \longrightarrow \rightarrow \mathbb {Q}_{+}$ is a map that assigns a positive weight to each transition; $\varGamma$ is a finite set of atomic propositions; and $\mathcal {L}: S \rightarrow 2^{\varGamma }$ is a labeling function.

#### Definition 8

A *timed run* of a WTS is an infinite sequence $r^t = (r(0), \tau (0))(r(1), \tau (1)) \ldots$, such that $r(0) \in S_0$, and for all $l \ge 1$, it holds that $r(l) \in S$ and (*r*(*l*), $\alpha (l)$, $r(l+1))$ $\in$ $\longrightarrow$ for a sequence of actions $\alpha (1) \alpha (2) \ldots$ with $\alpha (l) \in Act, \forall \ l \ge 1$. The *time stamps* $\tau (l)$, $l \ge 0$ are inductively defined as: 1) $\tau (0) = 0$; 2) $\displaystyle \tau (l+1) = \tau (l) + \mathfrak {t}(r(l), \alpha (l), r(l+1))$, $\forall \ l \ge 1.$ Every timed run $r^t$ generates a *timed word* $w^t = (w(0), \tau (0)) \ (w(1), \tau (1)) \ldots$ over the set $2^{\varGamma }$ where $w(l) = L(r(l))$, $\forall \ l \in \mathbb {N}$ is the subset of atomic propositions that are true at state *r*(*l*).

The syntax of *Metric Interval Temporal Logic (MITL)* over a set of atomic propositions $\varGamma$ is defined by the grammar:

$$\begin{aligned} \varphi := p \ | \ \lnot \varphi \ | \ \varphi _1 \wedge \varphi _2 \ | \ \Diamond _I \varphi \mid \square _I \varphi \mid \varphi _1 \ \mathcal {U}_I \ \varphi _2, \end{aligned}$$

where $p \in \varGamma$, and $\Diamond$, $\square$ and ${\mathcal {U}}$ are the eventually, always and until temporal operator, respectively; $I = [a, b] \subseteq \mathbb {Q}_{+}$ where $a, b \in [0, \infty ]$ with $a < b$ is a non-empty timed interval. The MITL formulas are interpreted over timed words like the ones produced by a WTS which is given in Definition 8.

#### Definition 9

(Souza and Prabhakar 2007; Ouaknine and Worrell 2005) Given a timed word $w^t = (w(0),\tau (0))(w(1),\tau (1)) \dots$, an MITL formula $\varphi$ and a position *l* in the timed word, the satisfaction relation $(w^t, l) \models \varphi$, for $l \ge 0$ (read $w^t$ satisfies $\varphi$ at position *l*) is inductively defined as follows:

$$\begin{aligned}&(w^t, l) \models p \Leftrightarrow p \in w(l), \\&(w^t, l) \models \lnot \varphi \Leftrightarrow (w^t, l) \not \models \varphi , \\&(w^t, l) \models \varphi _1 \wedge \varphi _2 \Leftrightarrow (w^t, l) \models \varphi _1 \ \text {and} \ (w^t, l) \models \varphi _2, \\&(w^t, l) \models \Diamond _I \varphi \Leftrightarrow \exists \ l' \ge l, \ \text {such that} \\&\quad \quad \quad (w^t, l') \models \varphi , \tau (l')-\tau (l) \in {I}, \\&(w^t, l) \models \square _I \varphi \Leftrightarrow \forall \ l' \ge l, \ \tau (l')-\tau (l) \in {I} \\&\quad \quad \quad \Rightarrow (w^t, l') \models \varphi , \\&(w^t, l) \models \varphi _1 \ \mathcal {U}_I \ \varphi _2 \Leftrightarrow \exists l' \ge l, \ \text {s.t. } (w^t, l') \models \varphi _2, \\&\quad \quad \quad \tau (l')-\tau (l) \in I \ \text {and} \ (w^t, l'') \models \varphi _1, \forall \ l \le l'' < l'. \end{aligned}$$

We say that a timed run $r^t = (r(0),\tau (0))(r(1),\tau (1)) \dots$ satisfies the MITL formula $\varphi$ (we write $r^t \models \varphi$) if and only if the corresponding timed word $w^t$ $= (w(0)$, $\tau (0))(w(1)$, $\tau (1)) \dots$ with $w(l) = L(r(l)), \forall \ l \ge 0$, satisfies the MITL formula ($w^t \models \varphi$).

*Timed Büchi Automata (TBA)* were originally introduced in Alur and Dill (1994); Bouyer (2009); Tripakis (2009). Let $\text {CL} = \{c_1$, $\ldots$, $c_{|\text {CL}|}\}$ be a finite set of *clocks*. The set of *clock constraints* $\varPhi (\text {CL})$ is defined by the grammar:

$$\begin{aligned} \phi := \top \mid \ \lnot \phi \ | \ \phi _1 \wedge \phi _2 \ | \ c \bowtie \psi , \end{aligned}$$

where $c \in \text {CL}$ is a clock, $\psi \in \mathbb {Q}_{+}$ is a clock constant and $\bowtie \ \in \{ <, >, \ge , \le , = \}$. A clock *valuation* is a function $\nu : \text {CL} \rightarrow \mathbb {Q}_{+}$ that assigns a value to each clock. A clock $c_l$ has valuation $\nu _l$ for $l \in \{1, \ldots , |\text {CL}|\}$, and $\nu = (\nu _1, \ldots , \nu _{|\text {CL}|})$. We denote by $\nu \models \phi$ the fact that the valuation $\nu$ satisfies the clock constraint $\phi$.

#### Definition 10

A *Timed Büchi Automaton* is a tuple $(Q, Q^{\text {init}}, \text {CL}, \mathrm{Inv}, E, \text {FS}, \varGamma , \mathcal {L})$ where *Q* is a finite set of locations; $Q^{\text {init}} \subseteq Q$ is the set of initial locations; $\text {CL}$ is a finite set of clocks; $\mathrm{Inv}: Q \rightarrow \varPhi (C)$ is the invariant; $E \subseteq Q \times \varPhi (\text {CL}) \times 2^C \times Q$ gives the set of edges of the form $e = (q, \mathfrak {g}, \text {RS}, q')$, where *q*, $q'$ are the source and target states, $\mathfrak {g}$ is the guard of edge *e* and $\text {RS}$ is a set of clocks to be reset upon executing the edge; $\text {FS} \subseteq Q$ is a set of accepting locations; $\varGamma$ is a finite set of atomic propositions; and $\mathcal {L}: Q \rightarrow 2^{\varGamma }$ labels every state with a subset of atomic propositions.

Any MITL formula $\varphi$ over $\varGamma$ can be algorithmically translated into a TBA with the alphabet $2^{\varGamma }$, such that the language of timed words (i.e. the set of all accepted timed words) that satisfy $\varphi$ is the language of timed words produced by the TBA (Alur et al. 1996; Maler et al. 2006; Ničković and Piterman 2010; Brihaye et al. 2017).

#### Definition 11

Given a WTS $\mathcal {T} =(S$, $S^{\text {init}}$, $\text {Act}$, $\longrightarrow$, $\mathfrak {t}$, $\varGamma$, $\mathcal {L})$, and a TBA $\mathcal {A} = (Q, Q^\text {init}, \text {CL}, \text {Inv}, E, \text {FS}, \varGamma )$ with $\text {CL}$ clocks. Then, their *product WTS*:

$$\begin{aligned} \widetilde{\mathcal {T}} = \mathcal {T} \circledast \mathcal {A} = (\widetilde{Q}, \widetilde{Q}^{\text {init}}, \rightsquigarrow , \widetilde{\mathfrak {t}}, \widetilde{\text {F}}, \varGamma , \widetilde{\mathcal {L}}), \end{aligned}$$

is defined as follows:

- $\widetilde{Q} = S \times Q$ is the set of states;
- $\widetilde{Q} ^{\text {init}} = S^{\text {init}} \times Q^{\text {init}}$ is the set of initial states;
- ${\rightsquigarrow }$ is the set of transitions where $(\widetilde{q}, \widetilde{q}') \in {\rightsquigarrow }$ iff:
  - $$\widetilde{q} = (s, q) \in \widetilde{Q} $$ and $$\widetilde{q}' = (s', q') \in \widetilde{Q} $$,
  - $$(s, \cdot , s') \in \longrightarrow $$, and
  - there exist $\mathfrak {g}, \gamma , \text {RS}$ such that $(q, \mathfrak {g}, \text {RS}, \gamma , q') \in E$ where $\gamma = \mathcal {L}(q')$;
- $\widetilde{\mathfrak {t}}(q, q') = \mathfrak {t}(s, s')$ if $(q, q') \in \rightsquigarrow$, is a map that assigns a positive weight to each transition;
- $\widetilde{F} = \{(s, q) \in \widetilde{Q} : q \in \text {FS}\}$ is a set of accepting states; and
- $\widetilde{\mathcal {L}}(s, q) = \mathcal {L}(s)$ is a labeling function.

## Problem formulation

### System model

Consider a team of *N* robots with labels $[N] {:}{=}\{1, \dots , N\}$ operating in a bounded workspace $\mathcal {W} \subseteq \mathbb {R}^n$. The robots are governed by the following kinematics and dynamics model:

1a $$\begin{aligned} \dot{x}_i&= v_i, \end{aligned}$$

1b $$\begin{aligned} \dot{v}_i&= f_i(x_i, v_i) + G_i u_i + \delta _i, \end{aligned}$$

where $x_i$, $v_i \in \mathbb {R}^{n}$ stands for the position/orientation and the linear/angular velocity of the robot $i \in [N]$, respectively; $f_i: \mathbb {R}^n \times \mathbb {R}^n \rightarrow \mathbb {R}^n$ is a known and continuously differentiable vector fields with $f_i(0, 0) = 0$ and $G_i \in \mathbb {R}^{n \times n}$; $u_i \in \mathbb {R}^n$ stands for the control input vector; and $\delta _i \in \mathbb {R}^n$ models the external disturbances and uncertainties. Consider also velocity constraints, input constraints as well as bounded disturbances:

$$\begin{aligned} v_i \in \mathcal {V}_i&{:}{=}\{v_i \in \mathbb {R}^n : \Vert v_i\Vert _2 \le \widetilde{v}_i \}, \\ u_i \in \mathcal {U}_i&{:}{=}\{u_i \in \mathbb {R}^n : \Vert u_i\Vert _2 \le \widetilde{u}_i \}, \\ \delta _i \in \varDelta _i&{:}{=}\{\delta _i \in \mathbb {R}^n : \Vert \delta _i\Vert _2 \le \widetilde{\delta }_i \}, \end{aligned}$$

where the constants $\widetilde{v}_i$, $\widetilde{u}_i$, $\widetilde{\delta }_i > 0$ are a priori given. The sets $\mathcal {V}_i$, $\mathcal {U}_i$ and $\varDelta _i$ are assumed to be connected sets with the origin as an interior point. Define the corresponding *nominal kinematics/dynamics* by:

2a $$\begin{aligned} \dot{\overline{x}}_i&= v_i, \end{aligned}$$

2b $$\begin{aligned} \dot{\overline{v}}_i&= f_i(\overline{x}_i, \overline{v}_i) + G_i \overline{u}_i, \end{aligned}$$

which are the real kinematics/dynamics for the case of $\delta _i = 0$.

#### Assumption 1

The linear systems $\dot{\overline{\eta }}_i = A_i \overline{\eta }_i + B_i \overline{u}_i$, where $\overline{\eta }_i {:}{=}[\overline{x}_i^\top , \overline{v}_i^\top ]^\top \in \mathbb {R}^{2n}$, that are the outcome of the Jacobian linearization of the nominal dynamics (2a)–(2b) around the equilibrium states $(x_i, v_i) = (0,0)$ are stabilizable.

#### Assumption 2

There exist strictly positive constants $\underline{G}_i$ such that:

3 $$\begin{aligned} \lambda _{\min }\left[ \frac{G_i + G_i^\top }{2}\right] \ge \underline{G}_i > 0, \ \forall i \in \mathcal {V}. \end{aligned}$$

#### Remark 1

Assumption 1 is required for the NMPC nominal stability to be guaranteed (Chen and Allgöwer 1998). Note also that in real-time robotic systems, the matrices $G_i$ usually represents the mass matrix of the robots which are always positive-definite. Thus, Assumption 2 is satisfied.

In the given workspace, there exist $Z \in \mathbb {N}$ disjoint Regions of Interest (RoI) labeled by $[Z] {:}{=}\{1,\dots , Z\}$. We assume that the RoI are modeled by balls, i.e., $\mathcal {R}_z {:}{=}\mathcal {M}(y_{z}, p_z)$, $Z \in \mathbb {N}$, where $y_{z}$ and $p_z > 0$ stand for the center and radius of RoI $\mathcal {R}_z$, respectively. Define also the union of RoI by

$$\begin{aligned} \mathcal {R} {:}{=}\bigcup _{z \in [Z]} \mathcal {R}_z. \end{aligned}$$

Due to the fact that we are interested in imposing safety constraints, at each time $t \ge 0$, the robot *i* is occupying a ball $\mathcal {M}(x_i(t), \mathfrak {r}_i)$ that covers its volume, where $x_i(t)$ and $\mathfrak {r}_i > 0$ are its center and radius, respectively. Moreover, in order to be able to impose transient constraints among the robots, we assume that each robot $i \in [N]$ has communication capabilities within a limited sensing range $d_i > 0$ such that:

4 $$\begin{aligned} d_i > \max _{i, j \in [N], i \ne j} \{\mathfrak {r}_i + \mathfrak {r}_j\}, \end{aligned}$$

The latter implies that each agent has sufficiently large sensing radius so as to measure the agent with the biggest volume, due to the fact that the agents’ radii are not the same. Define the set of robots *j* that are within the sensing range of agent *i* at time *t* as:

5 $$\begin{aligned} \mathcal {G}_i(t) {:}{=}\{j \in [N] \backslash \{i\} : \Vert x_i(t)-x_j(t)\Vert _2 < d_i\}. \end{aligned}$$

### Objectives

The goal of this paper is to design decentralized feedback control laws that steers the robots with dynamics as in (1a)–(1b) between RoI so that they obey individual high-level tasks given in MITL under transient constraints between them. Define the labeling functions:

6 $$\begin{aligned} \mathcal {L}_i: \mathcal {R} \rightarrow 2^{\varGamma _i}, \end{aligned}$$

which map each RoI with a subset of atomic propositions that hold true there. Note that some of the RoI may be assigned with labels that indicate *unsafe regions*, i.e., the robot is required to avoid visiting them (*safety specifications*).

#### Definition 12

A trajectory $x_i(t)$ of robot $i \in [N]$ is *associated with a timed run* $r_i^t$ $= (r_i(0)$, $\tau _i(0))$ $(r_i(1)$, $\tau _i(1))(r_i(2)$, $\tau _i(2))\ldots$, where $r_i(l) \in \mathcal {R}$, $\forall l \in \mathbb {N}$, is a sequence of RoI that the robot crosses, if the following hold:

1. $\tau _i(0) = 0$, i.e., the robot starts the motion at time $t = 0$;
2. $\mathcal {M}(x_i(\tau _i(0)), \mathfrak {r}_i) \subsetneq r_i(0)$, i.e., initially, the volume of the robot is entirely within the RoI $r_i(0) \in \mathcal {R}$;
3. $\mathcal {M}(x_i(\tau _i(l))$, $\mathfrak {r}_i) \subsetneq r_i(l)$, $\forall l \in \mathbb {N}$, i.e., the robot changes discrete state *as soon as* its entire volume is strictly contained in the corresponding RoI;
4. $$\tau _i(l+1) {:}{=}\tau _i(l) + \mathfrak {t}_i(r_i(l), r_i(l+1))$$, $$\forall l \in \mathbb {N}$$, where:
  7 $$\begin{aligned} \mathfrak {t}_i: \mathcal {R} \times \mathcal {R} \rightarrow \mathbb {Q}_{+}, \end{aligned}$$
  are functions that model the duration that the robot needs to be driven between regions $r_i(l)$ and $r_i(l+1)$.

#### Definition 13

A trajectory $x_i(t)$ *satisfies* an MITL formula $\varphi _i$ over the set of atomic propositions $\varGamma _i$, formally written as $x_i(t) \models \varphi _i$, $\forall t \ge 0$, if and only if there exists a timed run $r_i^t$ to which the trajectory $x_i(t)$ is associated, according to Definition 12, which satisfies $\varphi _i$.

#### Remark 2

We assume that the volume of each robot is covered by a ball. We further assume that the obstacles can be modeled by RoI that are also balls. Even if the volume of an agent and/or an obstacle is not a ball, it can be over-approximated by a ball.

### Problem statement

The problem considered in this paper is stated as follows:

#### Problem 1

Consider *N* robots governed by dynamics (1a)–(1b), covered by the balls $\mathcal {M}(x_i(t), \mathfrak {r}_i)$, operating in the workspace $\mathcal {W} \subseteq \mathbb {R}^{n}$ with sensing communication capabilities captured by the sets $\mathcal {G}_i$ as defined in (5). The workspace contains the RoI $\mathcal {R}_z$, $z \in [Z]$ modeled also by balls. Given task specification formulas $\varphi _i$ for each robot $i \in [N]$ expressed in MITL over the set of atomic propositions $\varGamma _i$ and labeling functions $L_i$ as in (6); then, design decentralized feedback control laws $u_i = \kappa _i(x_i, v_i) \in \mathcal {U}_i$, such that the robot trajectories in the workspace fulfill the MITL specifications $\varphi _i$, i.e., $x_i(t) \models \varphi _i$, $\forall t \ge 0$, according to Definition 12, while collision avoidance constraints are imposed among the robots, i.e.:

$$\begin{aligned} \Vert x_i-x_j\Vert _2 > \mathfrak {r}_i+\mathfrak {r}_j, \ \ \forall i \in [N], \ j \in [N] \backslash \{i\}. \end{aligned}$$

#### Remark 3

Note that Problem 1 constitutes a general problem due to the fact that the dynamics (1a)–(1b) arise in most robotic applications and transient constraints among the robots are taken into consideration.

## Problem solution

In this section, a systematic framework for solving Problem 1 is provided as follows:

1. In Sects. 4.1–4.2, decentralized feedback control laws that guarantee the transition between RoI in the given environments are provided. The laws consist of two components: an online control law which is the outcome of a DFHOCP solved at each timed step (Sect. 4.1); and an offline law which guarantees that the trajectories of the real system remain in a hyper-tube (Sect. 4.2).
2. Then, by using the outcome of Sect. 4.1, we abstract the dynamics (1a)–(1b) into a WTS for each robot, exploiting the fact that the timed runs in the WTS project onto associated trajectories according to Definition 12 (Sect. 4.3)
3. By invoking ideas from our previous work (Nikou et al. 2016), a controller synthesis procedure that provides a sequence of control laws that serve as solution to Problem 1 is consulted (Sect. 4.4)
4. Lastly, the computational complexity of the proposed framework is discussed in Sect. 4.5.

### Decentralized feedback control design—part I

Consider the robot *i* with dynamics (1a)–(1b) occupying a RoI $\mathcal {R}_{i,s} \in \mathcal {R}$ at time $\mathfrak {t}_{i,s} \ge 0$. The decentralized feedback control should guarantee that the robot is navigated towards a desired RoI $\mathcal {R}_{i,d} \in \mathcal {R}$, $\mathcal {R}_{i,d} \ne \mathcal {R}_{i,s}$ without intersection with any other RoI or other agents $j \in [N]$, $j \ne i$. Denote by $x_{i,d} \in \mathcal {R}_{i,d}$ the center of the RoI $\mathcal {R}_{i,s}$. Define the error vector:

$$\begin{aligned} e_i {:}{=}x_i - x_{i,d} \in \mathbb {R}^n, \ \ i \in [N], \end{aligned}$$

as well as the *uncertain error kinematics/dynamics* by:

8a $$\begin{aligned} \dot{e}_i&= v_i, \end{aligned}$$

8b $$\begin{aligned} \dot{v}_i&= f_i(e_i+x_{i,d}, v_i) + G_i u_i + \delta _i, \end{aligned}$$

The corresponding *uncertain nominal error kinematics/dynamics* are given by:

9a $$\begin{aligned} \dot{\overline{e}}_i&= \overline{v}_i, \end{aligned}$$

9b $$\begin{aligned} \dot{\overline{v}}_i&= f_i(\overline{e}_i+x_{i,d}, \overline{v}_i) + G_i \overline{u}_i. \end{aligned}$$

Consider the feedback control law:

10 $$\begin{aligned} u_i {:}{=}\overline{u}_i(\overline{e}_i, \overline{v}_i)+\kappa _i(e_i, v_i, \overline{e}_i, \overline{v_i}), \end{aligned}$$

which consists of a nominal control action $\overline{u}_i(\overline{e}_i, \overline{v}_i) \in \mathcal {U}_i$ and a state feedback laws $\kappa _i(e_i, v_i, \overline{e}_i, \overline{v_i})$. The control action $\overline{u}_i(\overline{e}_i, \overline{v}_i)$ is the outcome of a DFHOCP solved on-line at each sampling time step; the state-feedback law will be tuned off-line according to a procedure that will be presented thereafter.

Define the sets that capture the state constraints of each robot as:

$$\begin{aligned}&\mathcal {E}_i {:}{=}\Big \{ e_i(t) \in \mathbb {R}^n : \ \Vert e_i(t)+x_{i,d} - e_j(t)-x_{j,d}\Vert _2 > \\&\quad \mathfrak {r}_i + \mathfrak {r}_j + \tfrac{ \widetilde{\delta }_i}{\min \{\alpha _{i,1}, \alpha _{i,2}\}}, \ \forall j \in \mathcal {G}_i(t), \\&\quad \mathcal {M}(e_i(t)+x_{i,d}, \mathfrak {r}_i) \cap \{\mathcal {R} \backslash \{\mathcal {R}_{i,s} , \mathcal {R}_{i,d} \} \} = \emptyset \Big \}. \end{aligned}$$

The first constraint captures the fact that the robots should not collide with each other; the latter one, captures the fact that each robot needs to be navigated from RoI $\mathcal {R}_{i,s}$ to RoI $\mathcal {R}_{i,d}$ without intersecting with any other RoI of the workspace due to the fact that we are interested in imposing safety specifications.

#### Assumption 3

It is assumed that:

11 $$\begin{aligned} \mathfrak {r}_i + \tfrac{\widetilde{\delta }_i}{\min \{\alpha _{i,1}, \alpha _{i,2}\}} < p_z, \ \ \forall z \in [Z], \ \ i \in [N]. \end{aligned}$$

More specifically, (11) states that the radius of the ball that covers every robot plus the radius of the disturbance tube is smaller than the radius of any of the RoI of the workspace. As it will be shown later, this assumption is required in order to compute the time that a robot needs to be navigated between the RoI in the workspace.

Consider a sequence of sampling times $\{t_k\}$, $k \in \mathbb {N}$, with a constant sampling period $0< h < T$, where *T* stands for the finite prediction horizon. It holds that $t_{k+1} = t_k + h$, $\forall k \in \mathbb {N}$. It should be noted that both $t_k$ and *T* are multiples of *h*. At every discrete sampling time $t_k$ a DFHOCP is solved by each robot $i \in [N]$ as follows:

12a $$\begin{aligned}&\min \limits _{\overline{u}_i(\cdot )} \left\{ \Vert \overline{\xi }_i(t_k+T)\Vert ^2_{\scriptscriptstyle P_i} + \int _{t_k}^{t_k+T}\Big [ \Vert \overline{\xi }_i(\mathfrak {s})\Vert ^2_{\scriptscriptstyle Q_i} +\Vert \overline{u}_i(\mathfrak {s})\Vert ^2_{\scriptscriptstyle R_i} \Big ] d\mathfrak {s} \right\} \end{aligned}$$

12b $$\begin{aligned}&\text {subject to:} \nonumber \\&\dot{\overline{\xi }}_i(\mathfrak {s}) = \mathfrak {f}_i(\overline{e}_i(\mathfrak {s}), \overline{v}_i(\mathfrak {s}), \overline{u}_i(\mathfrak {s})), \end{aligned}$$

12c $$\begin{aligned}&\overline{\xi }_i(\mathfrak {s}) \in \overline{\mathcal {E}}_i \times \overline{\mathcal {V}}_i, \ \ \overline{u}_i(\mathfrak {s}) \in \overline{\mathcal {U}}_i, \ \ \forall \mathfrak {s} \in [t_k,t_k+T], \end{aligned}$$

12d $$\begin{aligned}&\overline{\xi }_i(t_k+T)\in \mathcal {F}_i. \end{aligned}$$

In the aforementioned optimal control problem we defined:

$$\begin{aligned} \overline{\xi }_i&{:}{=}[\overline{e}_i, \overline{v}_i]^\top \in \mathbb {R}^{2n}, \\ \mathfrak {f}_i(\overline{\xi }_i, \overline{u}_i)&{:}{=}\begin{bmatrix} \overline{v}_i \\ f_i(\overline{e}_i+x_{i,d}, \overline{v}_i) + \overline{u}_i \end{bmatrix}. \end{aligned}$$

The matrices $Q_i$, $P_i \in \mathbb {R}^{2n}$ and $R_i \in \mathbb {R}^n$ are positive definite weighting matrices. The sets $\mathcal {F}_i$ stand for the terminal sets that are used to enforce the stability of the nominal system (see Chen and Allgöwer 1998 for more details).

Hereafter, the sets $\overline{\mathcal {E}}_i$, $\overline{\mathcal {V}}_i$ and $\overline{\mathcal {U}}_i$ are explained. In order to guarantee that while the DFHOCP (12a)–(12d) is solved for the nominal dynamics (9a)–(9b), the real states $e_i$, $v_i$ and control inputs $u_i$ satisfy the corresponding state and input constraints $\mathcal {E}_i$, $\mathcal {V}_i$ and $\mathcal {U}_i$, respectively, the latter sets are appropriately modified as:

$$\begin{aligned} \overline{\mathcal {E}}_i&{:}{=}\mathcal {E}_i \ominus \varOmega _{i,1}, \\ \overline{\mathcal {V}}_i&{:}{=}\mathcal {V}_i \ominus \varOmega _{i,2}, \\ \overline{\mathcal {U}}_i&{:}{=}\mathcal {U}_i \ominus \left[ -k_i \circ \overline{\varOmega }_i \right] , \end{aligned}$$

with $\overline{\varOmega }_i {:}{=}\varOmega _{i,1} \oplus \varOmega _{i,2}$, $\varOmega _{i,1}$, $\varOmega _{i,2}$ as given in (16a), (16b), respectively, and $k_i$ to be defined later. This constitutes a standard constraints set modification technique adopted in tube-based NMPC frameworks (for more details see Yu et al. 2013). The advantage of the tube-based framework compared to other robust NMPC approaches is that the constraint tightening is performed offline and it does not depend on the length of the horizon. Algorithm 1 depicts the procedure of how the control law is calculated and applied to a real robot. This is a procedure of implementing the continuous-time tube-based NMPC in a real-time system that has been introduced in Yu et al. (2013).
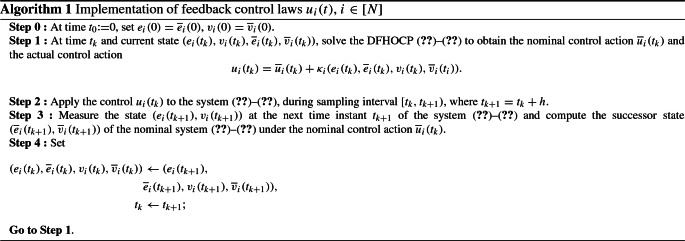


### Decentralized feedback control design—part II

For each agent $i \in [N]$ define by:

$$\begin{aligned} \mathfrak {e}_i&{:}{=}e_i - \overline{e}_i, \\ \mathfrak {v}_i&{:}{=}v_i - \overline{v}_i, \end{aligned}$$

the deviation between the real states $e_i$, $v_i$ of the uncertain system (8a)–(8b) and the states of the nominal system (9a)–(9b) with $\mathfrak {e}_i(0) = \mathfrak {v}_i(0) = 0$. It will be proven that the states $\mathfrak {e}_i$, $\mathfrak {v}_i$ remain invariant in certain compact sets. The dynamics of the states $\mathfrak {e}_i$ and $\mathfrak {v}_i$ are written as:

13a $$\begin{aligned} \dot{\mathfrak {e}}_i&= \dot{e}_i - \dot{\overline{e}}_i \nonumber \\&= v_i - \overline{v}_i \nonumber \\&= \mathfrak {v}_i, \end{aligned}$$

13b $$\begin{aligned} \dot{\mathfrak {v}}_i&= \dot{v}_i - \dot{\overline{v}}_i \nonumber \\&= f_i(e_i+x_{i,d}, v_i) - f_i(\overline{e}_i+x_{i,d}, \overline{v}_i) \nonumber \\&+ G_i u_i - G_i \overline{u}_i + \delta _i \nonumber \\&= g(e_i, \overline{e}_i, v_i, \overline{v}_i) + G_i \ (u_i - \overline{u}_i) +\delta _i, \end{aligned}$$

where the functions $g_i$ are defined by:

$$\begin{aligned} g_i(e_i, \overline{e}_i, v_i, \overline{v}_i) {:}{=}f_i(e_i+x_{i,d}, v_i) - f_i(\overline{e}_i+x_{i,d}, \overline{v}_i), \end{aligned}$$

and they are upper bounded by:

$$\begin{aligned}&\Vert g_i(e_i, \overline{e}_i, v_i, \overline{v}_i)\Vert _2 \le \Vert f_i(e_i+x_{i,d}, v_i) - f_i(\overline{e}_i+x_{i,d}, \overline{v}_i)\Vert _2 \\&\quad = \Vert f_i(e_i+x_{i,d}, v_i) - f_i(e_i+x_{i,d}, \overline{v}_i) \\&\quad \quad +f_i(e_i+x_{i,d}, \overline{v}_i)- f_i(\overline{e}_i+x_{i,d}, \overline{v}_i)\Vert _2 \\&\quad \le \Vert f_i(e_i+x_{i,d}, v_i) - f_i(e_i+x_{i,d}, \overline{v}_i)\Vert _2 \\&\quad \quad + \Vert f_i(e_i+x_{i,d}, \overline{v}_i)- f_i(\overline{e}_i+x_{i,d}, \overline{v}_i)\Vert _2 \\&\quad \le L_{v, i}\Vert v_i-\overline{v}_i\Vert _2 + L_{e, i}\Vert e_i+x_{i,d}-\overline{e}_i - x_{i,d}\Vert _2 \\&\quad = L_{v, i}\Vert v_i-\overline{v}_i\Vert _2 + L_{e, i}\Vert e_i-\overline{e}_i \Vert _2 \\&\quad = L_{v, i}\Vert \mathfrak {v}_i\Vert _2 + L_{e, i}\Vert \mathfrak {e}_i \Vert _2 \\&\quad \le L_i \left( \Vert \mathfrak {e}_i\Vert _2 + \Vert \mathfrak {v}_i\Vert _2\right) . \end{aligned}$$

The constants $L_{e,i}$, $L_{v,i}$ stand for the Lipschitz constants of functions $f_i$ with respect to the variable $e_i$ and $v_i$, respectively, and

$$\begin{aligned} L_i {:}{=}\max \{L_{e,i}, L_{v,i} \}, \ i \in [N]. \end{aligned}$$

#### Lemma 2

The state feedback laws designed by:

14 $$\begin{aligned} \kappa _i(e_i, \overline{e}_i, v_i, \overline{v}_i)&{:}{=}- k_i (e_i-\overline{e}_i)-k_i (v_i -\overline{v}_i), \ \ i \in [N], \end{aligned}$$

where $k_i$, $\rho _i > 0$ are chosen such that the following hold:

15 $$\begin{aligned} k_i> \frac{1}{\underline{G}_i} \left[ 1+(1+2\rho _i) L_i \right] , \ \ \rho _i > \frac{L_i}{2}, \end{aligned}$$

renders the sets:

16a $$\begin{aligned} \varOmega _{i, 1}&{:}{=}\left\{ \mathfrak {e}_i \in \mathbb {R}^{n} : \Vert \mathfrak {e}_i\Vert _2 \le \tfrac{\widetilde{\delta }_i}{\min \{\alpha _{i,1}, \alpha _{i,2}\}} \right\} , \end{aligned}$$

16b $$\begin{aligned} \varOmega _{i, 2}&{:}{=}\left\{ \mathfrak {v}_i \in \mathbb {R}^{n} : \Vert \mathfrak {v}_i\Vert _2 \le \tfrac{2 \ \widetilde{\delta }_i}{\min \{\alpha _{i,1}, \alpha _{i,2}\}} \right\} , \end{aligned}$$

RCI sets for the error dynamics (13a), (13b), according to Definition 3, where the constants $\alpha _{i,1}$, $\alpha _{i,2} > 0$ are defined by:

17 $$\begin{aligned} \alpha _{i,1} {:}{=}1 - \frac{L_i}{2 \rho _i}, \ \ \alpha _{i,2} {:}{=}k_i \ \underline{G}_i- 1 - (1+2\rho _i) L_i. \end{aligned}$$

#### Proof

A backstepping control methodology will be used (Krstic et al. 1995). The state $\mathfrak {v}_i$ in (13b) can be seen as virtual input to be designed such that the candidate Lyapunov function:

$$\begin{aligned} \mathfrak {L}_1(\mathfrak {e}_i) {:}{=}\frac{1}{2} \Vert \mathfrak {e}_i\Vert ^2_2, \end{aligned}$$

for the dynamical system (13a) is always decreasing. The time derivative of $\mathfrak {L}_1$ along the trajectories of system (13a) is given by:

$$\begin{aligned} \dot{\mathfrak {L}}_1(\mathfrak {e}_i)&= \mathfrak {e}_i^\top \dot{\mathfrak {e}}_i = \mathfrak {e}_i^\top \mathfrak {v}_i. \end{aligned}$$

Thus, by designing $\mathfrak {v}_i \equiv - \mathfrak {e}_i$, it yields that

$$\begin{aligned} \dot{\mathfrak {L}}_1(\mathfrak {e}_i)&= - \Vert \mathfrak {e}_i\Vert _2^2. \end{aligned}$$

Define the backstepping auxiliary errors $\zeta _{i, 1}$, $\zeta _{i, 2} \in \mathbb {R}^n$ by:

$$\begin{aligned} \zeta _{i, 1}&{:}{=}\mathfrak {e}_i, \\ \zeta _{i, 2}&{:}{=}\mathfrak {v}_i+\mathfrak {e}_i. \end{aligned}$$

Then, the auxiliary error dynamics are written as:

18a $$\begin{aligned} \dot{\zeta }_{i, 1}&= \dot{\mathfrak {e}}_i = \mathfrak {v}_i = \zeta _{i, 2}-\mathfrak {e}_i = - \zeta _{i, 1} + \zeta _{i, 2} \end{aligned}$$

18b $$\begin{aligned} \dot{\zeta }_{i,2}&= - \zeta _{i, 1} + \zeta _{i, 2} + g_i(\cdot ) + G_i \ (u_i - \overline{u}_i) + \delta _i, \end{aligned}$$

with:

18c $$\begin{aligned} \Vert g_i(\cdot )\Vert _2&\le L_i \ (\Vert \mathfrak {e}_i\Vert _2+\Vert \mathfrak {v}_i\Vert _2) \nonumber \\&= L_i \ (\Vert \zeta _{i,1}\Vert _2+\Vert \zeta _{i,1}-\zeta _{i,2}\Vert _2) \nonumber \\&\le 2L_i \ \Vert \zeta _{i,1}\Vert _2+ L_i \Vert \zeta _{i,2}\Vert _2, \end{aligned}$$

and $\zeta _{i,1}(0) = \zeta _{i,2}(0) = 0$. Define the stack vector $\zeta _i {:}{=}[\zeta _{i,1}^\top , \zeta _{i,2}^\top ]^\top \in \mathbb {R}^{2n}$ and consider the candidate Lyapunov function $\mathfrak {L}(\zeta _i) = \frac{1}{2}\Vert \zeta _i\Vert _2^2$ with $\mathfrak {L}(0) = 0$. The time derivative of $\mathfrak {L}$ along the trajectories of system (13a)–(13b) is given by:

$$\begin{aligned} \dot{\mathfrak {L}}(\zeta _i)&= \zeta _i^\top \dot{\zeta }_i \\&= \zeta _{i,1}^\top \dot{\zeta }_{i,1} + \zeta _{i,2}^\top \dot{\zeta }_{i,2} \\&= -\Vert \zeta _{i,1}\Vert ^2_2+\Vert \zeta _{i,2}\Vert ^2+\zeta _{i,2}^\top \ g_i(\cdot ) \\&\quad + \zeta _{i,2}^\top \ G_i \ (u_i - \overline{u}_i) + \zeta _{i,2}^\top \ \delta _i \\&\le -\Vert \zeta _{i,1}\Vert ^2_2+\Vert \zeta _{i,2}\Vert ^2+\Vert \zeta _{i,2}\Vert _2 \ \Vert g_i(\cdot )\Vert _2 \\&\quad + \zeta _{i,2}^\top \ G_i \ (u_i - \overline{u}_i) + \zeta _{i,2}^\top \ \delta _i \end{aligned}$$

By using (18c), the latter becomes:

$$\begin{aligned} \dot{\mathfrak {L}}(\zeta _i)&= -\Vert \zeta _{i,1}\Vert ^2_2+(L_i+1) \Vert \zeta _{i,2}\Vert _2^2 + 2 L_i \Vert \zeta _{i,1}\Vert _2\Vert \zeta _{i,2}\Vert _2 \\&\quad + \zeta _{i,2}^\top \ G_i \ (u_i - \overline{u}_i) + \zeta _{i,2}^\top \delta _i. \end{aligned}$$

By using Lemma 1 we have:

$$\begin{aligned} \Vert \zeta _{i,1}\Vert _2\Vert \zeta _{i,2}\Vert _2&\le \frac{\Vert \zeta _{i,1}\Vert ^2_2}{4 \rho _i} + \rho _i \Vert \zeta _{i,2}\Vert ^2 \\ \Rightarrow 2 L_i \Vert \zeta _{i,1}\Vert _2\Vert \zeta _{i,2}\Vert _2&\le \frac{L_i \Vert \zeta _{i,1}\Vert ^2_2}{2 \rho _i} + 2 \rho _i L_i \Vert \zeta _{i,2}\Vert ^2. \end{aligned}$$

with $\rho _i$ satisfying (15). Then, it holds that:

$$\begin{aligned} \dot{\mathfrak {L}}(\zeta _i)&\le - \left( 1 - \frac{L_i}{2 \rho _i} \right) \Vert \zeta _{i,1}\Vert ^2_2+\left[ 1+(1+2\rho _i) L_i\right] \Vert \zeta _{i,2}\Vert _2^2 \\&+ \zeta _{i,2}^\top \ G_i \ (u_i - \overline{u}_i) + \Vert \zeta _{i,2}\Vert \widetilde{\delta }_i. \end{aligned}$$

By designing $u_i - \overline{u}_i = -k_i\zeta _{i,2} = -k_i \mathfrak {e}_i-k_i \mathfrak {v}_i = -k_i (e-\overline{e}_i)-k_i (v_i-\overline{v}_i)$ which is the same with (14) and compatible with (10) we get:

$$\begin{aligned} \dot{\mathfrak {L}}(\zeta _i)&\le - \left( 1 - \frac{L_i}{2 \rho _i} \right) \Vert \zeta _{i,1}\Vert ^2_2+\left[ 1+(1+2\rho _i) L_i\right] \Vert \zeta _{i,2}\Vert _2^2 \\&\quad -k_i \ \zeta _{i,2}^\top \ G_i \ \zeta _{i,2} + \Vert \zeta _{i,2}\Vert \widetilde{\delta }_i. \end{aligned}$$

Writing the matrices $G_i$ as $G_i = \frac{G_i+G_i^\top }{2} + \frac{G_i-G_i^\top }{2}$ and taking into account that:

$$\begin{aligned}&y^\top \left( \frac{G_i-G_i^\top }{2} \right) y = 0, \ \ \forall y \in \mathbb {R}^n, \\&y^\top P y \ge \lambda _{\min }(P) \Vert y\Vert _2^2, \ \ \forall y \in \mathbb {R}^n, \ \ P \in \mathbb {R}^{n \times n}, \ \ P > 0, \end{aligned}$$

we obtain:

$$\begin{aligned}&\dot{\mathfrak {L}}(\zeta _i) \le \\&- \left( 1 - \frac{L_i}{2 \rho _i} \right) \Vert \zeta _{i,1}\Vert ^2_2+\left[ 1+(1+2\rho _i) L_i\right] \Vert \zeta _{i,2}\Vert _2^2 \\&-k_i \lambda _{\min } \left( \frac{G_i+G_i^\top }{2} \right) \Vert \zeta _{i,2}\Vert _2^2 + \Vert \zeta _{i,2}\Vert \widetilde{\delta }_i. \end{aligned}$$

By using Assumption 2 and (17), the latter becomes:

$$\begin{aligned} \dot{\mathfrak {L}}(\zeta _i)&\le - \alpha _{i,1} \Vert \zeta _{i,1}\Vert ^2_2-\alpha _{i,2} \Vert \zeta _{i,2}\Vert _2^2 + \Vert \zeta _{i,2}\Vert \widetilde{\delta }_i \\&\le - \min \{\alpha _{i,1}, \alpha _{i,2}\} \left( \Vert \zeta _{i,1}\Vert ^2_2+ \Vert \zeta _{i,2}\Vert ^2_2\right) + \Vert \zeta _{i,2}\Vert \widetilde{\delta }_i \\&= - \min \{\alpha _{i,1}, \alpha _{i,2}\} \Vert \zeta _{i}\Vert _2^2 + \Vert \zeta _{i,2}\Vert \widetilde{\delta }_i \\&= \Vert \zeta _{i}\Vert _2 \left[ - \min \{\alpha _{i,1}, \alpha _{i,2}\} \Vert \zeta _{i}\Vert _2 + \widetilde{\delta }_i \right] \end{aligned}$$

**Fig. 2:**
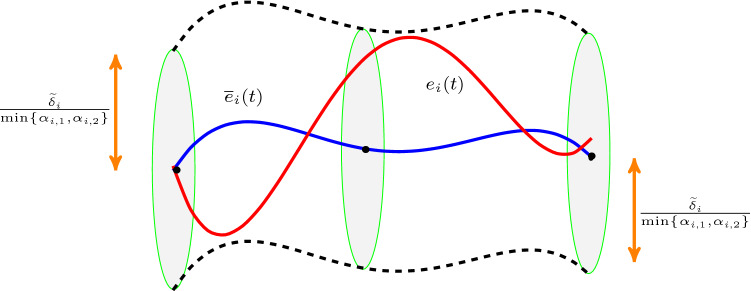
The hyper-tube centered along the trajectory $\overline{e}_i(t)$ (depicted by blue line) with radius $\tfrac{\widetilde{\delta }_i}{\min \{\alpha _{i,1}, \ \alpha _{i,2}\}}$. Under the proposed control law, the real trajectory $e_i(t)$ (depicted with red line) lies inside the hyper-tube for all times, i.e., $\Vert \mathfrak {e}_i(t)\Vert \le \tfrac{\widetilde{\delta }_i}{\min \{\alpha _{i,1}, \ \alpha _{i,2}\}}$, $\forall t \in \mathbb {R}_{\ge 0}$ (Color figure online)

Thus, $\dot{\mathfrak {L}}(\zeta _i) < 0$ when $\Vert \zeta _i\Vert _2 > \frac{\widetilde{\delta }_i}{\min \{\alpha _{i,1}, \alpha _{i,2}\}}$. Taking the latter into account and the fact that $\zeta _i(0) = 0$ we have that $\Vert \zeta _i(t)\Vert _2 \le \frac{\widetilde{\delta }_i}{\min \{\alpha _{i,1}, \alpha _{i,2}\}}$, $\forall t \ge 0$. Moreover, the following inequalities hold:

$$\begin{aligned} \Vert \mathfrak {e}_i\Vert _2&= \Vert \zeta _{i,1}\Vert _2 \le \Vert \zeta _i\Vert _2 \\ \Rightarrow \Vert \mathfrak {e}_i\Vert _2&\le \frac{\widetilde{\delta }_i}{\min \{\alpha _{i,1}, \alpha _{i,2}\}}, \ \ \forall t \ge 0, \end{aligned}$$

and:

$$\begin{aligned} \big |\Vert \mathfrak {e}_i\Vert _2 - \Vert \mathfrak {v}_i\Vert _2\big |&\le \Vert \mathfrak {e}_i + \mathfrak {v}_i\Vert _2 \le \Vert \zeta _{i,2}\Vert _2 = \Vert \zeta _i\Vert _2 \\ \Rightarrow \Vert \mathfrak {v}_i\Vert _2&\le \frac{2 \ \widetilde{\delta }_i}{\min \{\alpha _{i,1}, \alpha _{i,2}\}}, \ \ \forall t \ge 0, \end{aligned}$$

which leads to the conclusion of the proof. $\square$

The aforementioned result states that real trajectories $e_i(t)$, $v_i(t)$ will belong to a hyper-tubed which is centered along the nominal trajectories $\overline{e}_i(t)$, $\overline{v}_i(t)$. The tubes’ radii are $\frac{\widetilde{\delta }_i}{\min \{\alpha _{i,1}, \alpha _{i,2}\}}$ and $\frac{2 \widetilde{\delta }_i}{\min \{\alpha _{i,1}, \alpha _{i,2}\}}$, respectively, as it is depicted in Fig. 2.

#### Remark 4

It should be noted that the volume of the hyper-tubes depends on the upper bound of the disturbances $\delta _i$, the Lipschitz constants $L_i$ and the constants $\underline{G}_i$. By tuning the gains $k_i$ and $\rho _i$ as in (15), (17) appropriately, the volume of the tubes can be adjusted. However, these gains cannot be set arbitrarily high due to the fact that the robots have limited actuation resources which are captured by the upper bound of the control input. The higher the upper bound of the control input is, the smaller the volume of the tube can be set.

By using (10), the closed-loop system is written as:

19a $$\begin{aligned} \dot{e}_i&= v_i, \end{aligned}$$

19b $$\begin{aligned} \dot{v}_i&= f_i(e_i+x_{i, d}, v_i)+\overline{u}_i(\overline{e}_i, \overline{v}_i) - k_i (e_i-\overline{e}_i) \nonumber \\&-k_i (v_i -\overline{v}_i)+\delta _i. \end{aligned}$$

Due to the fact that Problem 1 imposes transient constraints between the agents (collision avoidance) and the agents have communication capabilities within the sensing range $d_i$ as given in (4)–(5), we adopt here the decentralized procedure depicted in Algorithm 2 and explained hereafter. Assume that each agent knows its labeling number in the set [*N*]. After each sampling time $t_k$, $\forall k \ge 0$ that agent *i* solves its own DFHOCP and obtains the estimated open-loop trajectory $\overline{\xi }_i(\mathfrak {s})$, $\mathfrak {s} \in [t_k, t_k+T]$, it transmits it to all agents $j \in \mathcal {G}_i(t_k)$, $j \ne i$, i.e., to agents that are within its sensing radius at time $t_k$. Then, agents’ $j \in \mathcal {G}_i(t_k)$, $j \ne i$ hard constraints $\overline{\mathcal {E}}_{j}$ are updated by incorporating the predicted trajectory of agent *i*, i.e., $\overline{\xi }_i(\mathfrak {s})$, $\mathfrak {s} \in [t_k, t_k+T]$. Among all agents $j \in \mathcal {G}_i(t_k)$, the one with higher priority, i.e., smaller labeling number in the set [*N*], solves its own DFHOCP (for example, agent 2 has higher priority than agents 3, 4, $\dots$). This *sequential procedure* is continued until all agents $i \in [N]$ solve their own DFHOCP, and then the sampling time is updated.

In other words, each time an agent solves its own individual optimization problem, it knows the (open-loop) state predictions that have been generated by the solution of the optimization problem of all agents within its sensing range at that time, for the next *T* time units. These pieces of information are required, as each agent’s trajectory is constrained not by constant values, but by the trajectories of its associated agents through time: at each solution time $t_k$ and within the next *T* time units, an agent’s predicted configuration at time $\mathfrak {s} \in [t_k, t_k + T]$ needs to be constrained by the predicted configuration of its neighboring and perceivable agents (agents within its sensing range) at the same time instant $\mathfrak {s}$, so that collisions are avoided. We assume that the above pieces of information are *always available*, *accurate* and can be exchanged without delay. We will show thereafter that by adopting the aforementioned sequential communication procedure, and given that at $t = 0$ the DFHOCP (12a)–(12d) of all agents are feasible, the agents are navigated to the desired RoI, while all distance and input constraints imposed by Problem 1 are satisfied.

#### Remark 5

It should be noted that the constraint sets $\overline{\mathcal {E}}_i$, $i \in [N]$ in (12c) depend on the estimated open-loop trajectories $\overline{e}_i(\mathfrak {s})$ and $\overline{e}_j(\mathfrak {s})$ for all $i \in [N]$, $j \in \mathcal {G}(t_k)$, with $\mathfrak {s} \in [t_k, t_k+T]$. Moreover, they are updated when each robot has received the transmitted trajectories by its neighbors.

#### Remark 6

By considering a real-time scenario where the state vector $\overline{\xi }$ is comprised of 12 real numbers encoded by 4 bytes the overall downstream bandwidth required by each robot is:

$$\begin{aligned} BW_d = 12 \times 32\ \text {[bits]} \times |\mathcal {G}_i(t_k)| \times \dfrac{T}{h} \times f\ [\text {sec}^{-1}]. \end{aligned}$$

Given a conservative sampling time $f = 100$ Hz and a horizon of $\dfrac{T}{h} = 100$ time steps, the wireless protocol IEEE 802.11n-2009 (a standard for present-day devices) can accommodate up to

$$\begin{aligned} |\mathcal {G}_i(t_k)| = \dfrac{600\ [\text {Mbit}\cdot \text {sec}^{-1}] }{12\times 32[\text {bit}]\times 10^4 [\text {sec}^{-1}]} \approx 16 \cdot 10^2 \text { robots}, \end{aligned}$$

within the range of one robot. We deem this number to be large enough for practical applications of the proposed approach.

The following theorem guarantees the navigation of the agents between RoI and thereafter we will propose algorithms computing the corresponding transition times.

#### Theorem 1

Suppose that Assumptions 1–3 hold. Suppose that the robots start at time $\mathfrak {t}_{i,s} \ge 0$ from the RoI $\mathcal {R}_{i,s}$ and they need to be navigated to RoI $\mathcal {R}_{i,d}$ for every $i \in [N]$. Suppose also that at time $\mathfrak {t}_{i,s}$ the DFHOCP (12a)–(12d) sequentially solved by all the robots $i \in [N]$, is feasible. Then, the proposed decentralized feedback control law (10), (14), renders the closed-loop system (19a)–(19b) of each robot $i \in [N]$ Input to State Stable with respect to $\delta _i(t) \in \varDelta _i$.

#### Proof

The proof of the theorem consists of two parts:

**Feasibility Analysis**: It can be shown that recursive feasibility is established and it implies subsequent feasibility. The proof of this part is similar to the feasibility proof of (Filotheou et al. (2018), Theorem 2, Sec. 4, p. 12).

**Convergence Analysis**: Recall that:

$$\begin{aligned} e_i&= x_i-x_{i, d}, \ \ \mathfrak {e}_i = e_i-\overline{e}_i, \ \ \mathfrak {v}_i = v_i-\overline{v}_i. \end{aligned}$$

Then, we get:

$$\begin{aligned} \Vert x_i(t)-x_{i, d}\Vert _{2}&\le \Vert \overline{e}_i(t)\Vert _{2} + \Vert \mathfrak {e}_i(t)\Vert _{2}, \\ \Vert v_i(t)\Vert _{2}&\le \Vert \overline{v}_i(t)\Vert _{2} + \Vert \mathfrak {v}_i(t)\Vert _{2}, \end{aligned}$$

which, by using the fact that:

$$\begin{aligned} \Vert \overline{e}_i\Vert _2 \le \Vert \overline{\xi }_i\Vert _{2}, \ \ \Vert \overline{v}_i\Vert _2 \le \Vert \overline{\xi }_i\Vert _{2}, \end{aligned}$$

as well as the bounds from (16a), (16b), become:

20a $$\begin{aligned} \Vert x_i(t)-x_{i, d}\Vert _{2}&\le \Vert \overline{\xi }_i(t)\Vert _{2} + \tfrac{ \widetilde{\delta }_i}{\min \{\alpha _1, \alpha _2\}}, \end{aligned}$$

20b $$\begin{aligned} \Vert v_i(t)\Vert _{2}&\le \Vert \overline{\xi }_i(t)\Vert _{2} + \tfrac{2 \widetilde{\delta }_i}{\min \{\alpha _1, \alpha _2\}}, \ \ \forall t \ge 0. \end{aligned}$$

Since only the nominal system dynamics (9a)–(9b) are used for the online computation of the control action $\overline{u}_i(\mathfrak {s}) \in \overline{\mathcal {U}}_i$, $\mathfrak {s} \in [t_k, t_k+T]$ through the DFHOCP (12a)–(12d), by invoking nominal NMPC stability results found on Chen and Allgöwer (1998), it can be shown that there exist class $\mathcal {KL}$ functions $\varvec{\beta }_i$, such that:

21 $$\begin{aligned} \Vert \overline{\xi }_i(t)\Vert \le \varvec{\beta }_i(\Vert \overline{\xi }_i(\mathfrak {t}_{i,s})\Vert _2, t), \ \ \forall t \in \mathbb {R}_{\ge 0}. \end{aligned}$$

By combining (20a)–(20b) with (21) we get:

22a $$\begin{aligned} \Vert x_i(t)-x_{i, d}\Vert _{2}&\le \varvec{\beta }_i(\Vert \overline{\xi }_i(\mathfrak {t}_{i,s})\Vert _2, t) + \tfrac{ \widetilde{\delta }_i}{\min \{\alpha _1, \alpha _2\}}, \end{aligned}$$

22b $$\begin{aligned} \Vert v_i(t)\Vert _{2}&\le \varvec{\beta }_i(\Vert \overline{\xi }_i(\mathfrak {t}_{i,s})\Vert _2, t) + \tfrac{2 \widetilde{\delta }_i}{\min \{\alpha _1, \alpha _2\}}. \end{aligned}$$

for every $t \in \mathbb {R}_{\ge 0}$. The latter inequalities leads to the conclusion of the proof. $\square$

### Discrete system abstraction

Theorem 1 implies that for each robot $i \in [N]$ with kinematics/dynamics as in (1a), (1b), starting from the RoI $\mathcal {R}_{i, s}$ at time $\mathfrak {t}_{i, s}$, is driven by the controller (10) towards a desired RoI $\mathcal {R}_{i, d}$, while all state, input and transient constraints are satisfied. Hereafter, we provide an algorithm for constructing the WTS of each agent. By observing (22a) and taking into account Assumption 3, it holds that there exists a time instant $\mathfrak {t}_{i,d}$ such that the volume of robot *i* will be included strictly within the RoI $\mathcal {R}_{i,d}$. Furthermore, due to the fact that we have knowledge of the nominal dynamics and the MITL tasks $\varphi _i$ are independent for each robot, for the computation of the time $\mathfrak {t}_{i,d}$ an offline computer simulation of the DFHOCP (12a)–(12d) with state constraints as:

23 $$\begin{aligned} \widetilde{\mathcal {E}}_i&\,{:}{=}\,\Big \{e_i(t) \in \mathbb {R}^n : \mathcal {M}(e_i(t)+x_{i,d}, \mathfrak {r}_i) \nonumber \\&\cap \{\mathcal {R} \backslash \{\mathcal {R}_{i,s} , \mathcal {R}_{i,d} \} \} \Big \} \ominus \varOmega _{i,1}, \end{aligned}$$

is conducted. In particular, (23) captures constraints regarding the navigation of robot *i* from RoI $\mathcal {R}_{i,s}$ to RoI $\mathcal {R}_{i,d}$ without intersecting with any other RoI of the workspace. It should be noted that if any collision is about to occur in real-time when the robots are executing the on-line control actions, the transition time between the RoI will be different. In order to overcome the aforementioned issue, we will provide thereafter an algorithm that monitors the collision offline and updates the transition times appropriately. Then, the process of computing $\mathfrak {t}_{i,d}$ is described in Algorithm 3. The abstraction that captures the dynamics of each robot into a WTS is given through the following definition.
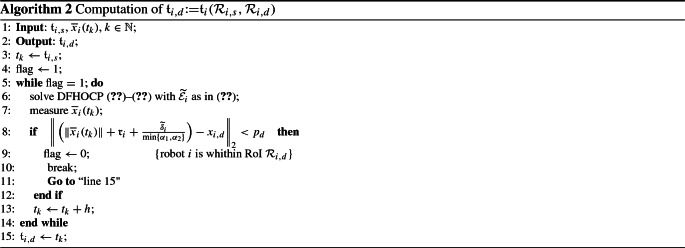

**Fig. 3:**
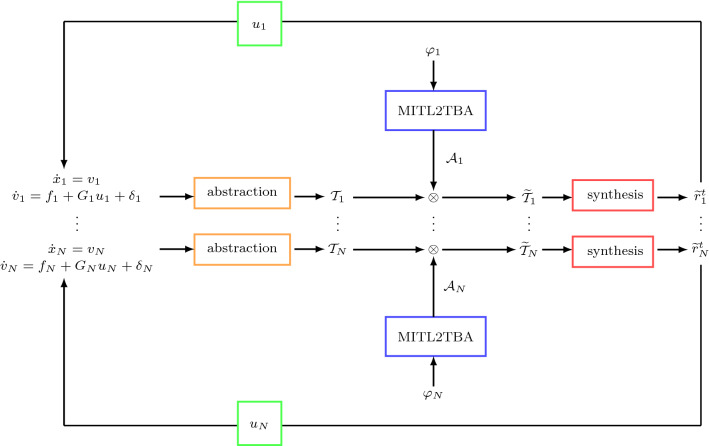
A graphic illustration of the proposed framework

#### Definition 14

The motion of robot *i* in the workspace $\mathcal {W}$ is modeled by the WTS

$$\begin{aligned} \mathcal {T}_i = (S_i, S_{i}^{\text {init}}, \mathrm{Act_i}, \longrightarrow , \mathfrak {t}_i, \varGamma _i, L_i), \end{aligned}$$

where:

- $S_i = \mathcal {R} = \displaystyle \bigcup \nolimits _{z \in [Z]} \mathcal {R}_z$ is the set of states of the robot that contains all the RoI of the workspace $\mathcal {W}$;
- $S_{i}^{\text {init}} \subseteq S_i$ is a set of initial states defined by the robot’ s initial position $x_i(0)$ in the workspace;
- $\mathrm{Act_i}$ is the set of actions containing the union of all feedback controllers (10) which can navigate the robot *i* between RoI;
- $\longrightarrow _i \subseteq S_i \times \mathrm{Act_i} \times S_i$ is the transition relation. We say that $(\mathcal {R}_{i, s}, u_i, \mathcal {R}_{i, d}) \in \longrightarrow _i$, with $\mathcal {R}_{i, s}$, $\mathcal {R}_{i, d} \in \mathcal {R}$ with $\mathcal {R}_{i, s} \ne \mathcal {R}_{i, d}$ if there exist feedback control law $u_i \in \mathrm{Act_i}$ as in (10) which can drive the robot from the region $\mathcal {R}_{i, s}$ to the region $\mathcal {R}_{i, d}$ without intersecting with any other RoI of the workspace;
- $\mathfrak {t}_i$ is the time weight as given in (7) and it is computed by Algorithm 2;
- $\mathcal {L}_i$ is the labeling function as given in (6);
- and $\varGamma _i$ is the set of atomic propositions imposed by Problem 1.

The aforementioned WTS of each robot allows us to work directly at the discrete level and design a sequence of feedback controllers as in (10) that solve Problem 1. By construction, each timed run produced by the WTS $\mathcal {T}_i$, where the notion of timed run is given in Definition 8, is associated with the trajectory $x_i(t)$ of the system (1a)–(1b), as given in Definition 12. Hence, if a timed run of $\mathcal {T}_i$ of each robot $i \in [N]$ satisfying the given MITL formula $\varphi _i$ is found, a desired timed word of the original system, and hence a trajectory $x_i(t)$ that is a solution to Problem 1 is found.

### Control synthesis

Figure 3 depicts a framework under which a sequence of feedback control laws $u_i(x_i, v_i)$ of each robot that guarantees the satisfaction of the MITL formula $\varphi _i$ can be computed. First, a TBA $\mathcal {A}_i$ that accepts all the timed runs satisfying the specification formula $\varphi _i$ is constructed. Second, a product between the WTS $\mathcal {T}_i$ given in Definition 14 and the TBA $\mathcal {A}_i$ is computed which gives the product WTS $\widetilde{\mathcal {T}}_i$. By performing graph search to the product WTS $\widetilde{\mathcal {T}}_i$, a timed run that satisfies the MITL formula $\varphi$ can be found. For more details regarding the control synthesis procedure we refer to our previous work (Nikou et al. 2016, 2018).

In view of Algorithm 3, (23) and the offline plan computation, it is possible that while each agent is executing online its individual actions and transits between RoI, there might be a cluster of agents that avoid collision between each other. In such a scenario, the online feedback control law avoids the possible collisions, but the navigation time between the RoI will have been different that the one computed by Algorithm 3. In order to resolve this, we propose an offline collision detection algorithm (see Algorithm 4) which detects the cluster of agent that will avoid potential collision when the plan of each agent is executed and updates the transition times between RoI of each agent appropriately.

More specifically, the input to Algorithm 4 is the transition times of each agent and the output is the updated realistic transition times denoted by $\mathfrak {t}^{\text {real}}_{i,d}$ as well as the formula bounds relaxation. The function $\text {computePlanAgent}(i)$ computes the sequence of RoI that agent *i* needs to follow in order to satisfy the formula. The function $\text {executePlanAgent}(i)$ executes a simulated plan for each agent. Then, by using a monitoring function

$$\begin{aligned} \text {collisionClusterMonitoring}, \end{aligned}$$

the cluster of the agents that are colliding can be detected. Then, we need to update the transition times of each of the colliding agents by a term which models the time duration of the maneuvering that the corresponding agent is performing in order to avoid the collision. This time is denoted in Algorithm 4 by $T_{\text {maneuver}}^{i}$. By finding the maximum of the aforementioned times, the time bounds of the MITL formula of each agent are relaxed. The function $\text {relaxBounds}(\varphi _i, \max _i)$ updates each formula time interval of the form [*a*, *b*], $a > b \ge 0$, to $[a, b+\max _i]$.

#### Proposition 1

The solution that it is obtained from the controller synthesis procedure provides a sequence of feedback control laws $u_i(x_i, v_i)$ as in (10) that guarantees the satisfaction of the formula $\varphi$ of the robot governed by dynamics as in (1a)–(1b), thus, providing a solution to Problem 1.


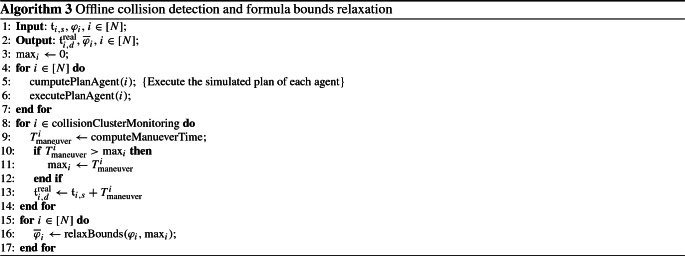


### Complexity analysis

The proposed framework consists of the computational complexity of the following steps:

- **C1:** the computational complexity of the offline construction of WTS $\widetilde{\mathcal {T}}_i$ and graph search. In particular, the graph search is performed over the product WTS $\widetilde{\mathcal {T}}_i$ which has $|S_i| \cdot |Q_i|$ number of states, i.e., the multiplication between the states of the WTS (number of RoI of the workspace) and the number of states of the TBA. The complexity of the Dijskstra algorithm that is used for the graph search is: $\mathcal {O} \Big ( |S_i| \cdot |Q_i| + |\text {edges}| \log \big (|S_i| \cdot |Q_i| \big ) \Big )$, where $|\text {edges}|$ is the number of edges of the product WTS $\widetilde{\mathcal {T}}_i$.
- **C2:** Algorithm 2 is an offline computer simulation and the computational complexity is the same with the complexity of a nominal NMPC algorithm;
- **C3:** Algorithm 3 is an offline computer simulation of collision detection which scales with the number of agents;
- **C4:** the DFHOCP (12a)–(12d) is the only online commutation of the proposed framework and has the same complexity with the nominal NMPC algorithm (quadratic programming optimization technique).

By taking into account that **C1** is standard in timed verification, and the fact that **C2**, **C4** have the same complexity with nominal NMPC, and **C3** is a computer simulation that scales with the number of agents, the proposed approach is scalable with the number of agents.

## Experimental setup and results

In this section the efficacy of the proposed framework via a real-time experiment employing $N = 3$ Nexus 10011 mobile robots is validated. The experiment was conducted at Smart Mobility Lab (SML) (see Fig. 1 and Smart Mobility Lab (SML) (n.d.)). By controlling the speed of each wheel, the Nexus Robot 10011 is able to move forward, backward, left, and right. The robot can also rotate clockwise and counterclockwise. In other word, it has three degrees of freedoms, i.e moving forward/backward, moving left/right and rotation. By combining the three degree of freedom, the Nexus Robot is able to move towards any direction. SML provides a motion capture system (MoCap) with 12 cameras spread across the lab. The MoCap provides the robot state vector, including pose, orientation as well as linear and angular velocities at frequency of 100Hz. The software implementation of the proposed control strategy was conducted in C++ under Robot Operating System (ROS) (Quigley et al. 2009). Moreover, the optimization algorithms described in this chapter are implemented by employing the NLopt Optimization library found in Johnson (2009).

**Fig. 4:**
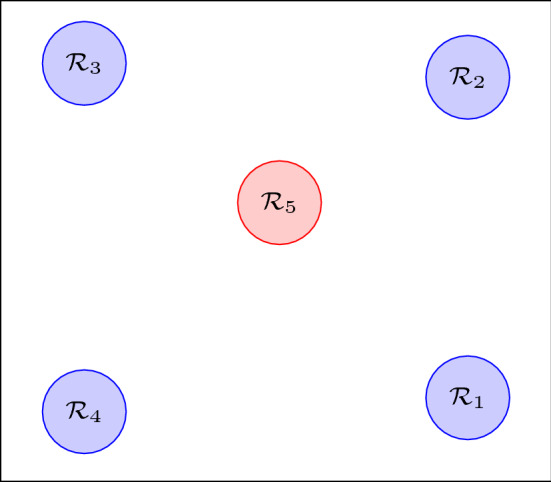
A panoramic view of the workspace with the 5 RoI (Color figure online)

The state of each robot is $x_i = [x_{i,1}, x_{i,2}, x_{i,3}]^\top$ where $x_{i,1}$, $x_{i,2}$ indicate the position of the robot and $x_{i,3}$ its orientation. The workspace that the robots can operate in as well as a panoramic view of it is depicted in Figs. 1 and 4, respectively. The workspace is captured by the set:

$$\begin{aligned} \mathcal {W} {:}{=}\{w \in \mathbb {R}^2 : |w_{k}| \le 2.5, \ \ k \in \{1,2\}\}, \end{aligned}$$

and it contains 5 RoI which are divided as follows:

- the RoI $\mathcal {R}_z$, $z \in \{1, 2, 3, 4\}$ depicted with blue color in Fig. 4 which stand for the RoI that the robots are required to visit. The RoI $\mathcal {R}_z$, $z \in \{1, 2, 3, 4\}$ map into the atomic propositions that model missions for each robot;
- the RoI $\mathcal {R}_5$ depicted with red color in Fig. 4 stands for an unsafe region that the robots should avoid collision with. It holds that $\mathcal {L}_i(\mathcal {R}_5) = \{ \text {obs} \}$ for every $i \in [N]$.

The control input constraints of each robot are set to:

$$\begin{aligned} \mathcal {U}_i = \{u_{i} \in \mathbb {R}^3 : |u_{i,k}| \le 0.15, \ \ k \in \{1,2,3\} \}, \ \ i \in [N], \end{aligned}$$

where $u_{i,1}$, $u_{i,2}$ stand for the linear velocities and $u_{i,3}$ stands for the angular velocity. The ball that covers the volume of each robot has radius $\mathfrak {r}_i = 0.4 \text {m}$ for every $i \in [N]$. The sensing radius of each robot is $\mathfrak {d}_i = 2 \text {m}$. The robots 1, 2 and 3 are initially place in the ROI $\mathcal {R}_1$, $\mathcal {R}_2$ and $\mathcal {R}_3$, respectively. The set of atomic propositions of each robot is given by:

$$\begin{aligned} \varPi _1&= \{\text {obs}, \text {mission}_{11}, \text {mission}_{13}\}, \\ \varPi _2&= \{\text {obs}, \text {mission}_{22}, \text {mission}_{24}\}, \\ \varPi _3&= \{\text {obs}, \text {mission}_{33}, \text {mission}_{32}\}, \end{aligned}$$

with the corresponding labeling functions:

$$\begin{aligned} \mathcal {L}_1(\mathcal {R}_1)&= \{\text {mission}_{11}\}, \ \mathcal {L}_1(\mathcal {R}_2) = \emptyset , \\ \mathcal {L}_1(\mathcal {R}_3)&= \{\text {mission}_{13}\}, \mathcal {L}_1(\mathcal {R}_4) = \emptyset , \\ \mathcal {L}_2(\mathcal {R}_1)&= \emptyset , \ \mathcal {L}_2(\mathcal {R}_2) = \{\text {mission}_{22}\}, \\ \mathcal {L}_2(\mathcal {R}_3)&= \emptyset , \mathcal {L}_2(\mathcal {R}_4) = \{\text {mission}_{24}\}, \\ \mathcal {L}_3(\mathcal {R}_1)&= \emptyset , \ \mathcal {L}_3(\mathcal {R}_2) = \{\text {mission}_{32}\}, \\ \mathcal {L}_3(\mathcal {R}_3)&= \{\text {mission}_{33}\}, \ \mathcal {L}_3(\mathcal {R}_4) = \emptyset . \end{aligned}$$

The desired MITL tasks are set to:

$$\begin{aligned} \varphi _1&= \square _{[0,120]} \{\lnot \text {obs}\} \wedge \Diamond _{[10,25]} \{ \text {mission}_{13}\} \\&\wedge \Diamond _{[30,45]} \{ \text {mission}_{11}\}, \\ \varphi _2&= \square _{[0,120]} \{\lnot \text {obs}\} \wedge \Diamond _{[25,45]} \{ \text {mission}_{22}\} \\&\wedge \Diamond _{[50,80]} \{ \text {mission}_{24}\}, \\ \varphi _3&= \square _{[0,120]} \{\lnot \text {obs}\} \wedge \Diamond _{[30,45]} \{ \text {mission}_{33}\} \\&\wedge \Diamond _{[60,75]} \{ \text {mission}_{32}\}, \end{aligned}$$

respectively. The prediction horizon is chosen $T = 2.0 \sec$. The tube of each robot is given by the set:

$$\begin{aligned} \varOmega _{i} = \left\{ \mathfrak {e}_i : \Vert \mathfrak {e}_i\Vert \le \frac{\widetilde{\delta }_i}{k_i} \right\} . \end{aligned}$$

The NMPC gains are set to:

$$\begin{aligned} Q_i = P_i = R_i = 0.5 I_3, \ \ i \in [N]. \end{aligned}$$

By using Algorithm 3 and Algorithm 4, the total transition times of the navigation of the robots between the RoI of the workspace are computed as follows:

$$\begin{aligned} \mathfrak {t}_1(\mathcal {R}_1, \mathcal {R}_3)&= \mathfrak {t}_1(\mathcal {R}_3, \mathcal {R}_1) = 18, \\ \mathfrak {t}_2(\mathcal {R}_2, \mathcal {R}_4)&= \mathfrak {t}_2(\mathcal {R}_4, \mathcal {R}_2) = 20, \\ \mathfrak {t}_3(\mathcal {R}_2, \mathcal {R}_3)&= \mathfrak {t}_3(\mathcal {R}_3, \mathcal {R}_2) = 16. \end{aligned}$$

**Fig. 5:**
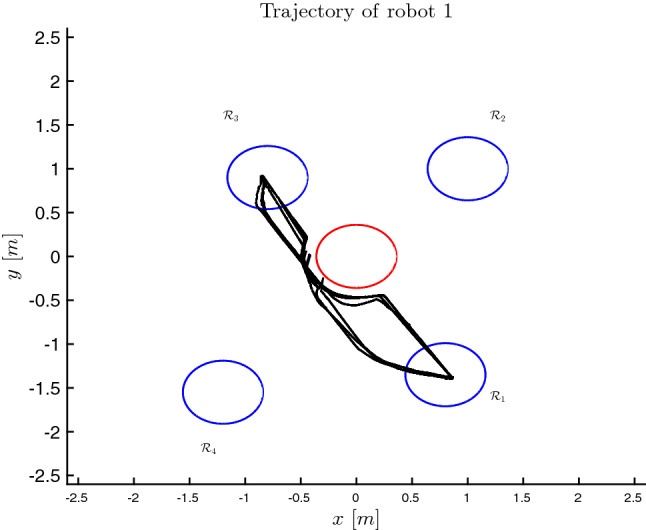
The trajectory of robot 1 in the workspace

**Fig. 6:**
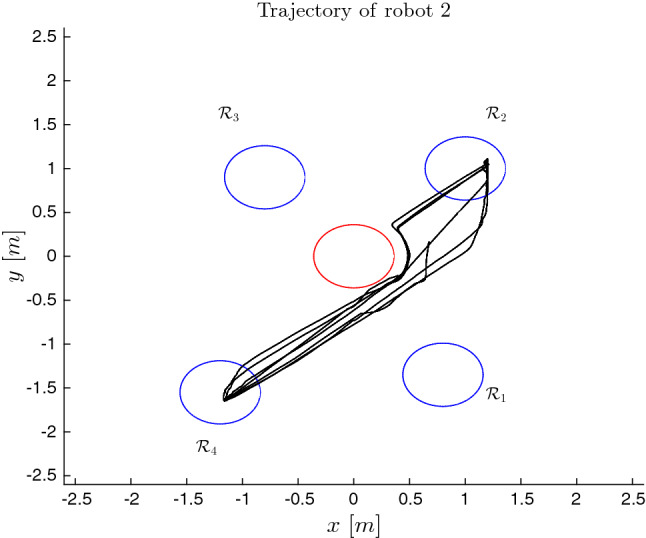
The trajectory of robot 2 in the workspace

**Fig. 7:**
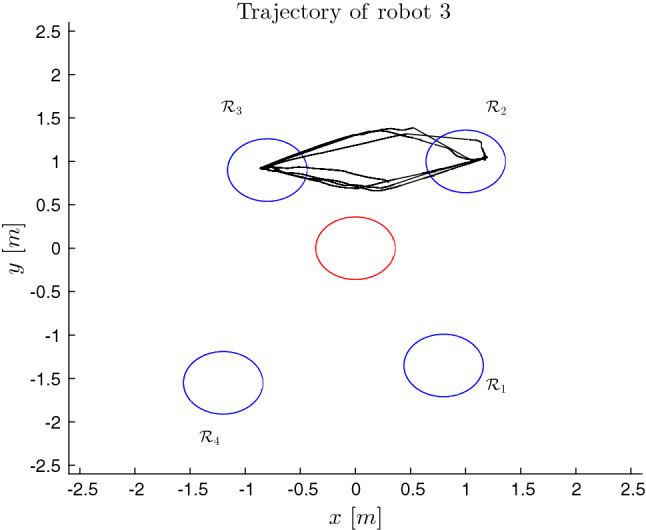
The trajectory of robot 3 in the workspace

By using the proposed framework, we find a sequence of runs of each agent that fulfills the given MITL task. The sequence of runs maps into a sequence of feedback control laws that the robot execute online and fulfill the given tasks. By online executing the proposed plan, the trajectories of the robots in the workspace are depicted in Figs. 5, 6 and 7.

**Video:** A video demonstrating the experiment of this section can be found in the following link: https://www.youtube.com/watch?v=9ZNVlEjKZ9g.

## Conclusions and future work

In this paper, a scalable framework for time-constrained planning of multi-robot systems has been proposed. Considering *N* robots operating in a bounded workspace which contains RoI, assigned with tasks given in MITL, a framework for efficiently designing decentralized feedback control laws that guarantee the satisfaction of the corresponding tasks has been provided. The controllers are the outcome of DFHOCP solved by each robot at each sampling time and form the actions of the WTS. By proposing high-level controller synthesis algorithms, a sequence of feedback laws for each robot can be designed. The approach is scalable since the local products are computed offline and only the DFHOCP of each robot is computed online which has complexity similar with the nominal NMPC framework. Future research directions will be devoted towards incorporating event-triggered strategies between he robots in order to save valuable actuation and sensing resources.
